# Lethality of Zinc Oxide Nanoparticles Surpasses Conventional Zinc Oxide via Oxidative Stress, Mitochondrial Damage and Calcium Overload: A Comparative Hepatotoxicity Study

**DOI:** 10.3390/ijms23126724

**Published:** 2022-06-16

**Authors:** Xingyao Pei, Haiyang Jiang, Gang Xu, Cun Li, Daowen Li, Shusheng Tang

**Affiliations:** 1 Department of Pharmacology and Toxicology, College of Veterinary Medicine, China Agricultural University, Yuanmingyuan West Road No.2, Haidian District, Beijing 100193, China; b20203050366@cau.edu.cn (X.P.); haiyang@cau.edu.cn (H.J.); 2 Tianjin Key Laboratory of Agricultural Animal Breeding and Healthy Husbandry, Department of Animal Pharmacy, College of Animal Science and Veterinary Medicine, Tianjin Agricultural University, Jinjing Road No.22, Xiqing District, Tianjin 300384, China; xugangayu@163.com (G.X.); hhlicun@163.com (C.L.); 3 Tianjin Key Laboratory of Biological Feed Additive Enterprise, S&E Burgeoning Biotechnology (Tianjin) Co., Ltd., Tianjin 300383, China; 4 State Key Laboratory of Medicinal Chemical Biology and Tianjin Key Laboratory of Molecular Drug Research, College of Pharmacy, Nankai University, Haihe Education Park, Tongyan Road No.38, Tianjin 300353, China

**Keywords:** zinc oxide nanoparticles, ZnO, hepatotoxicity, RNA-seq, oxidative stress

## Abstract

Zinc oxide nanoparticles (ZnO NPs) with high bioavailability and excellent physicochemical properties are gradually becoming commonplace as a substitute for conventional ZnO materials. The present study aimed to investigate the hepatotoxicity mechanism of ZnO NPs and traditional non-nano ZnO particles, both in vivo and in vitro, and identify the differences in their toxic effects. The results showed that the extent and conditions of zinc ion release from ZnO NPs were inconsistent with those of ZnO. The RNA-seq results revealed that the expression quantity of differentially expressed genes (DEGs) and differentially expressed transcripts (DETs) affected by ZnO NPs was more than in ZnO, and the overall differences in genes or transcripts in the ZnO NPs group were more pronounced than in the ZnO group. Furthermore, the cell inactivation, oxidative stress, mitochondrial damage, and intracellular calcium overload induced by ZnO NPs were more serious than ZnO in HepG2 cells. Moreover, compared with traditional ZnO, the rat liver damage induced by ZnO NPs was more significant, with evidence of higher AST and ALT levels, weaker antioxidant capacity, and more serious histopathological damage (*p* < 0.05). In summary, the hepatotoxicity of ZnO NPs was more serious than that of conventional ZnO, which is helpful to understand the hepatotoxicity mechanism of Zn compounds in different states and improve the risk assessment of novel nano ZnO products in a variety of applications.

## 1. Introduction

It is common knowledge that ZnO has been used widely in the field of both industrial and everyday chemical use as a Zn-derived chemical additive. However, with the development of nanotechnology, ZnO NPs have gradually been taken up as a new substitute for ZnO materials in industrial production, biomedicine, clinical therapy, food processing and environmental control [1]. ZnO NPs are one of the most widely used nanomaterials after SiO_2_ and TiO_2_; they are prepared from ZnO by a variety of methods, such as physical, chemical and biological template techniques [2]. Nevertheless, nano-sized ZnO possesses unique advantages in terms of volume effect, surface effect and quantum size effect, compared with the traditional ZnO noumenon [3,4]. The previous literature has revealed that ZnO NPs mainly induce acute and subchronic toxicity via different administration modes and models. The LD_50_ level was calculated as 0.3 mg/kg after a single injection of ZnO NPs into the tail vein of mice [5]. A single intraperitoneal injection of ZnO NPs in mice showed an LD_50_ of 299.9 mg/kg, accompanied by histopathological damage to multiple organs [6]. In a subchronic trial, inflammatory injury occurred in the stomach, pancreas, eyes and prostate of SD rats after continuous intragastric administration of 125 mg/kg ZnO NPs for 90 d [7]. As the research further developed, the teratogenic, carcinogenic and mutagenic properties of ZnO NPs were also discovered. Fetal malformation, skeletal dysplasia and spinal insufficiency were found after the administration of 100 mg/kg selenium (Se)-doped ZnO NPs for 14 consecutive days from the fifth day of pregnancy [8]. In addition, an intraperitoneal injection of ZnO NPs at a concentration of 245.26 mg/kg induced mutagenicity in chickens, which was characterized by micronucleus formation, binucleation and heteromorphism in red blood cells [9]. Moreover, the potential carcinogenic risk presented by ZnO NPs was also evaluated, which showed that the MutT homolog 1 (MTH1), an enzyme specifically expressed by cancer cells, was upregulated by exposure to ZnO NPs, and was involved in regulating the long-term effects of carcinogenicity induced by ZnO NPs, including proliferation, independent migration and invasion [10]. Furthermore, ZnO NPs could trigger toxicity in various target organs, including hepatorenal toxicity, cardiotoxicity, neurotoxicity, immunotoxicity and reproductive toxicity. After the stimulation of ZnO NPs, serum urea and creatinine levels in mice were obviously increased, the renal tubule epithelial cells were exfoliated, and the glomerulus contracted and ruptured [11]. It was shown that the cardiotoxicity in rats was triggered by oral administration of 600 mg/kg ZnO NPs for 5 d, with the up-regulation of creatine kinase-MB, intracellular calcium overload, and DNA damage [12]. After an intraperitoneal injection of ZnO NPs at a dose of 5.6 mg/kg, the blood-brain barrier was damaged, resulting in a disorder of nerve cell arrangement, neuronal degeneration, and the loss of nistenite [13]. It was also identified in the rat spleen and thymus exposed to ZnO NPs that the expression of malondialdehyde (MDA) was increased, and the levels of IL-1β, TNF-α, TLR4 and TLR6 were enhanced [14]. After the intragastric administration of 100 mg/kg ZnO NPs for 3 d, the Zn element had accumulated in mouse reproductive organs, also leading to testicular weight loss in male mice, as a result of oxidative stress and apoptosis. The Shh pathway-mediated apoptosis in ovaries and the Caspase-related damage in uteri were also observed in female mice [15]. The above evidence is summarized in Table 1; they prove that although ZnO NPs possess their own unique application value, improper use may cause toxicity to various organs and bring threats to humans and animals.

Early investigations into these health risks identified the liver as one of the main target organs for ZnO NP accumulation. The distribution of ZnO NPs in mouse and rat tissues through the routes of oral, intravenous, intraperitoneal injection and inhalation were measured in 2015, indicating that ZnO NPs were mainly distributed in the liver, kidney and lung [16]. Another pharmacokinetic study confirmed that the liver was the first organ showing ZnO NP uptake in Wistar rats. The accumulated amount of ZnO NPs in the liver reached a peak at 30 min, i.e. 20%, which decreased to 11% within 24 h and dropped to 3.3% after 7 d [17]. As a result of 90 days of intragastric treatment with 500 mg/kg of ZnO NPs, Zn concentration in the livers of 5-week-old rats increased in a dose-dependent manner [18]. Within 60 minutes of intravenous injection, ZnO NPs were found deposited in the liver and spleen, reaching peak values on day 6 and day 1, respectively, suggesting a relationship between the accumulation level and macrophage phagocytosis [5]. Besides this, the hepatotoxic damage produced by ZnO NPs was also revealed. In the BALB/C mice stimulated by intraperitoneal injections of 50 and 100 mg/kg ZnO NPs for 14 consecutive days, the liver exhibited plate disappearance, accompanied by parenchymal cell denaturation, hepatocyte enlargement, nuclear pyknosis and Kupffer cell increase [19]. After 4 days of treatment with 1 mg/L ZnO NPs in a marine environment, inflammatory cell infiltration, hepatocyte necrosis and lipid vacuolation were verified in juvenile fish [20]. As is consistent with this pathological evidence, long-term exposure to ZnO NPs could enhance the signs of liver inflammatory injury, manifested as the significant up-regulation of IL-8 secretion [21], as well as promote inflammatory liver macrophage damage through the toll-like receptor-mediated MAPK signaling pathway [22]. Alanine aminotransferase (ALT), aspartate aminotransferase (AST) and alkaline phosphatase (ALP), the markers of hepatotoxicity, were also elevated by ZnO NPs, and the gene expressions of the drug metabolism enzyme genes, cyp3a11, cyp2c29 and ugt2b, were down-regulated [23,24,25,26,27]. Recent evidence also determined the crucial position of oxidative stress and mitochondrial damage in the activation of the transduction pathway or DNA damage induced by ZnO NPs. The DCFH-DA and JC-1 fluorescence assay confirmed that reactive oxygen species (ROS) accumulation and mitochondrial damage in HepG2 cells were evoked by 7.2 µg/mL of ZnO NPs–ferulate acid (FAC) complex and, furthermore, led to the overexpression of endogenous apoptosis markers, such as Bax, cleaved-caspase-3 and cleaved-PARP [28]. It has been demonstrated that ZnO NPs could be absorbed by lettuce in the natural environment. In a simulated digestion test in vitro, the 0.151 mM ZnO NPs found in lettuce digestion solution promoted ROS generation in human hepatocytes (HL7702), and the mitochondrial membrane potential (MMP) was reduced to 52.4% of the control group [29]. Another study proved that the level of MDA was increased by 50 mg/kg ZnO NPs exposure, along with decreased catalase (CAT) and glutathione S-transferase (GST), while ROS production, motivated by ZnO NPs, was alleviated by N-acetylcysteine (NAC) [30]. To be brief, the liver has been deemed an important target organ of toxicity induced by ZnO NPs; they are a driving force of oxidative stress and mitochondrial damage that we are unable to ignore.

**Figure 1:**
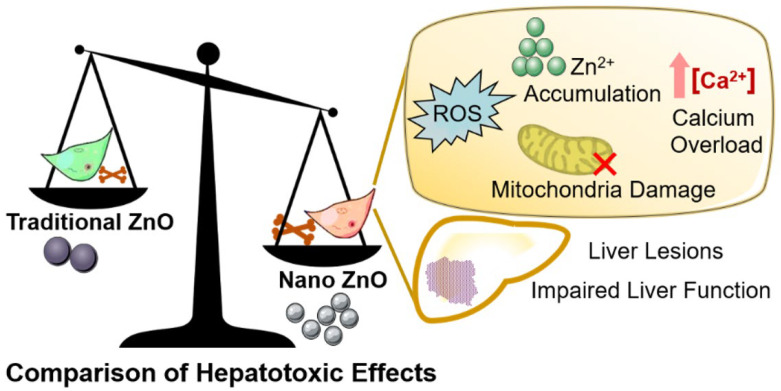
The hepatotoxicity mechanism and the differences between Nano ZnO and traditional ZnO.

Based on previous evaluations [31], the transport mechanism and toxic effects of ZnO NPs with the equivalent element content in vivo and in vitro were more complex than that of unitary ZnO. The evidence in vivo demonstrated that ZnO NPs mainly existed in the form of Zn ions and nanoparticles in the intestine [32,33,34]. The ZIPs and ZnTs protein families were the primary receptors for Zn ion absorption and transport, and the metallothionein family (MTs) were regulators for Zn ion storage and release. However, the intestinal absorption of ZnO NPs relies on endocytosis [35,36,37,38]. The evidence in vitro showed that ZnO NPs mainly enter cells in two states, zinc ions and nanoparticles; the former affected enzyme balance, the expression of transcription factors and changes to the signaling pathways, while the latter may produce overlapping reactions or other additional effects, including oxidative stress, mitochondrial damage, Ca^2+^ overload, etc. Another important point was that ZnO NPs were easily converted into Zn ions in the lysosome, thus disrupting lysosome homeostasis [39]. In addition to the different transport mechanisms, the nanometer form was found to increase the biotoxicity of ZnO in some toxicological models. After non-toxic doses of ZnO were replaced with ZnO NPs, the toxic effects immediately became visible, initiating apoptosis in mice microglia via the ERK and AKT pathways [40]. The toxicity of ZnO NPs and ZnO on astrocytes was also compared; the results showed that both of them could destroy mitochondrial function, while the ROS production and caspase activity of astrocytes exposed to ZnO NPs was more significant than traditional ZnO. At a dose of 40 μg/mL, the number of viable cells in the ZnO NPs group obviously decreased, while for ZnO, no significant effect on cell proliferation was discovered [41]. There were also signs that the hepatotoxicity of conventional ZnO was likely to be enhanced. In subacute toxicity tests, the 50 mg/L ZnO NPs showed higher bio enrichment and brought more severe histopathological changes and more obvious oxidative stress in carp, compared to ZnO, while the target tissues of the ZnO NPs were identified as the liver and gills, the latter mainly accruing in the intestine [42]. Moreover, ZnO NPs displayed a higher absorption rate in mice than ZnO, resulting in more conspicuous liver hepatological injury [43]. However, a systematic comparison of hepatotoxicity caused by ZnO NPs and ZnO is still lacking. This paper focused on the liver, the most important target organ for the accumulation and toxicity of ZnO NPs, to clarify the liver toxicity mechanism of ZnO NPs and traditional non-nano ZnO through in vivo and in vitro evaluation, while the predicted comparisons of the molecular mechanisms are presented in Figure 1. Further investigation was devoted to anatomizing the influence of nano effects on hepatotoxicity, in order to improve the risk assessment and reasonable application of ZnO nano products in various industries.

## 2. Results

### 2.1. Physicochemical Properties and Comparisons of Zn Ions

**Figure 2:**
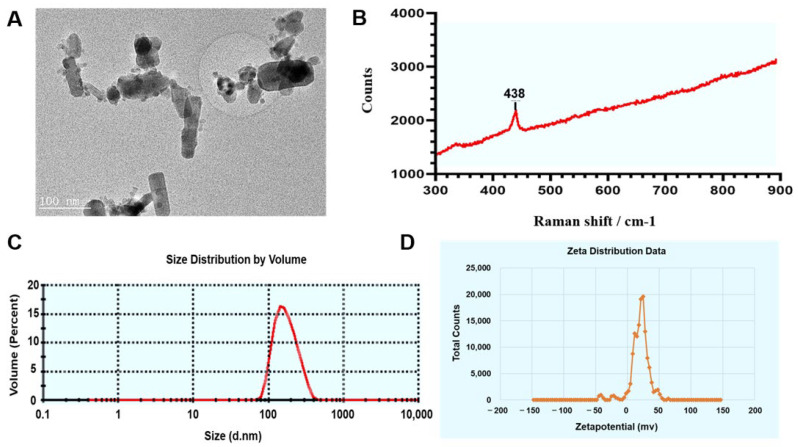
Characterization of ZnO NPs. (**A**) ZnO NPs were observed using TEM (Bar = 100 nm). (**B**) Determination of ZnO NPs by Raman spectroscopy. (**C**) The separate coefficient, the degree of polymerization and the hydrodynamic size of ZnO NPs were evaluated by dynamic light scattering. (**D**) The zeta potential of ZnO NPs was determined by using a zeta potential analyzer.

**Figure 3:**
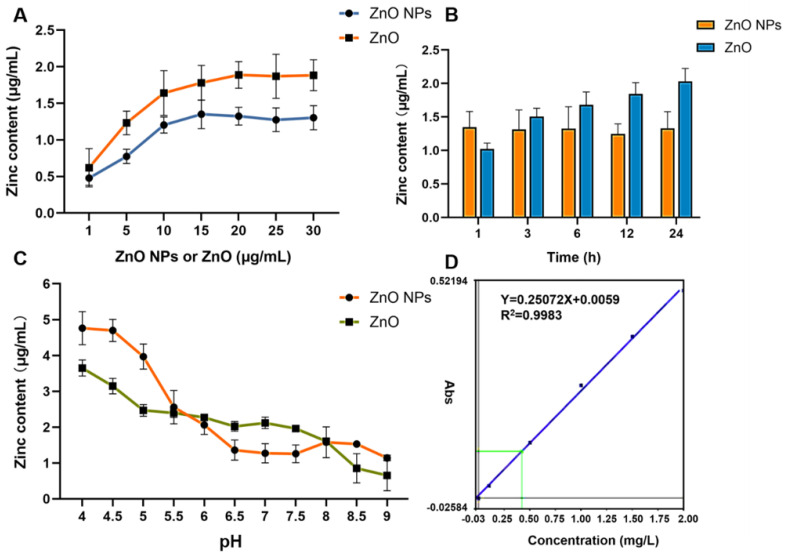
Determination of zinc ion release in cell culture medium. (**A**) Detection of zinc ion release from different concentrations of ZnO NPs and ZnO in cell culture medium, using FAAS. (**B**) Detection of zinc ion release from ZnO NPs and ZnO in cell culture medium at different times, using FAAS. (**C**) Detection of zinc ion release from ZnO NPs and ZnO in different pH cell culture mediums, using FAAS. (**D**) Standard curve of FAAS for the determination of zinc. Data represent the mean ± SD.

As shown in the transmission electron microscopy (TEM) image in Figure 2A, the cross-section was observed to be round, long, elliptical or other different shapes, confirming the inhomogeneous size and shape of the ZnO NPs. The ZnO NPs exhibited dispersion, with some slight contact and agglomeration. After testing the Raman spectra, the sample was identified as a single component with a characteristic peak of 438 cm^−1^ (Figure 2B). Furthermore, as described for the dynamic light scattering tests, we found that the size distribution of the ZnO NPs suspension was homogeneous, characterized by the peak pattern shown in Figure 2C. The hydrate particle size and the dispersion coefficient were recorded as 169.2 ± 61.74 nm and 0.135, respectively. On further analysis, it was determined that the zeta potential of ZnO NPs was 20.00 ± 6.49 mv, under neutral conditions in Milli-Q water (Figure 2D). According to the FAAS data in Figure 3A–D, the release of Zn ions in the cell culture fluid kept rising as the concentration of the ZnO NPs and ZnO increased within the range from 1 μg/mL to 15 μg/mL. Conversely, the ZnO had a stronger release ability in spite of being at the same concentration, being characterized by a higher platform compared with ZnO NPs (Figure 3A). Notably, it can also be inferred from Figure 3B that the time taken by ZnO NPs to reach the release platform was almost negligible, while the ZnO gradually released Zn icons before the end of 24 h, and the concentration of Zn ions that collected from 3 h to 24 h was greater than in the ZnO NPs group. Surprisingly, in acidic conditions (pH ≤ 5.5), the amount of Zn ions released in the ZnO NPs group surpassed that of the ZnO group (Figure 3C), revealing that the pH value provided the turning point for this comparison test.

### 2.2. Transcriptome Contrast

**Figure 4:**
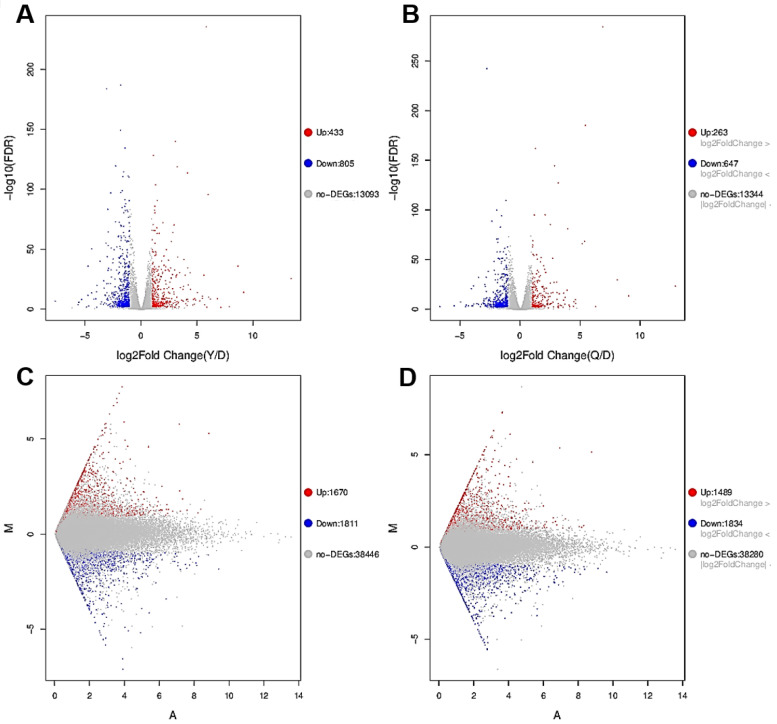
RNA-seq analysis of DEGs and DETs of ZnO NPs and ZnO in a HepG2 cell. (**A**) Volcano map of DEGs between ZnO NPs (20 µg/mL) and control group. (**B**) Volcano map of DEGs between ZnO (20 µg/mL) and control group. (**C**) MA map of DETs between ZnO NPs (20 µg/mL) and control group. (**D**) MA map of DETs between ZnO (20 µg/mL) and control group. The red dots represent genes that were significantly upregulated, and the blue dots represent genes that were significantly downregulated (*n* = 3).

**Figure 5:**
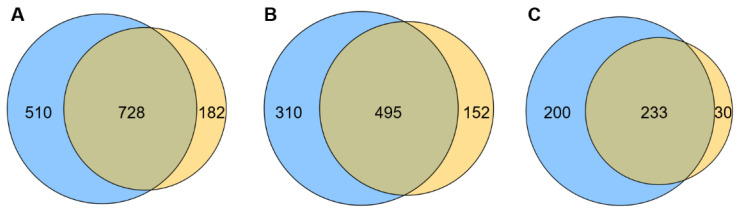
Wayne analysis of DEGs. (**A**) DEGs, shared by ZnO NPs and control group, ZnO and the control group. (**B**) Upregulated genes, shared by ZnO NPs and the control group, and ZnO and the control group. (**C**) Downregulated genes, shared by the ZnO NPs and the control group, and ZnO and the control group.

**Figure 6:**
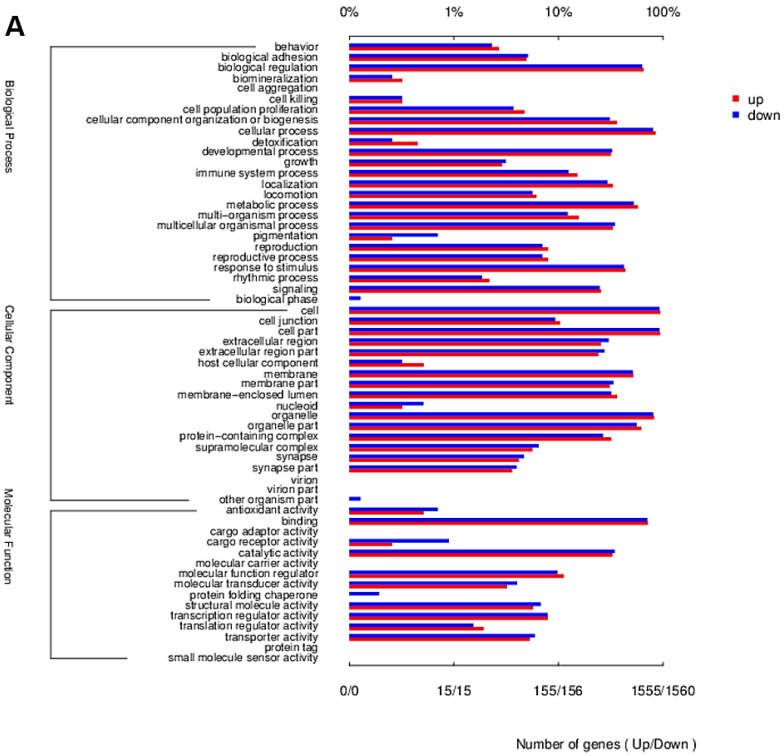

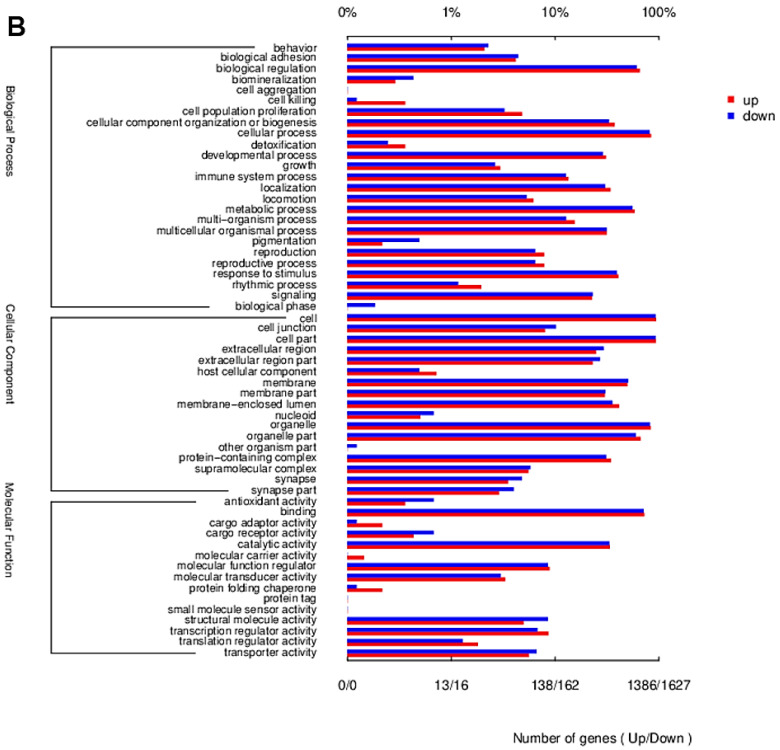

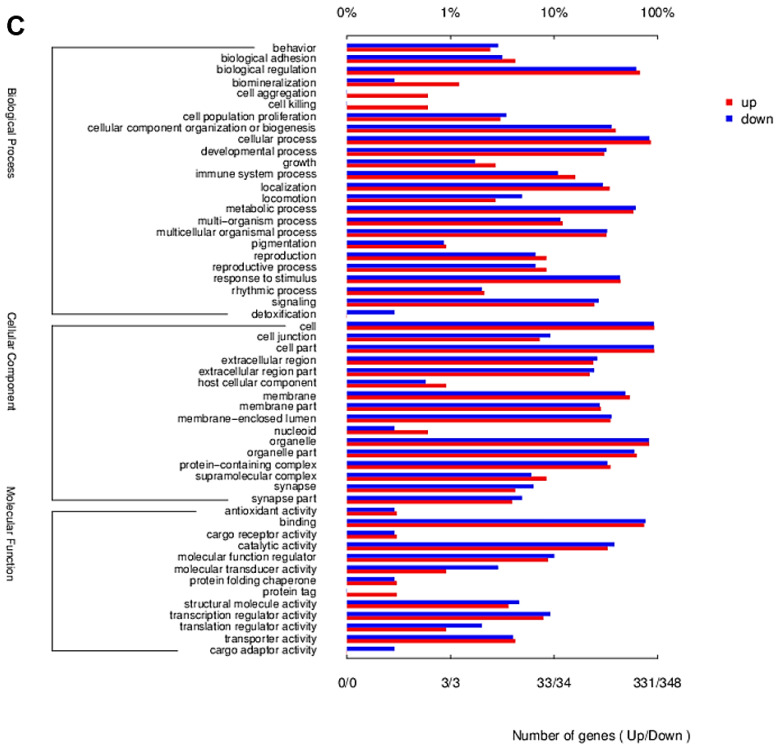
GO functional enrichment analysis of DETs. (**A**) GO functional enrichment analysis of DETs between ZnO NPs and the control group. (**B**) GO functional enrichment analysis of DETs between ZnO and the control group. (**C**) GO functional enrichment analysis of DETs between ZnO NPs and the ZnO group.

The overall distribution of differentially expressed genes (DEGs) and differentially expressed transcripts (DETs) in HepG cells, stimulated by 20 µg/mL ZnO NPs and 20 µg/mL ZnO, were visualized. As shown in Figure 4A,B, 1238 genes were significantly changed by ZnO NPs compared with the control group, including 433 upregulated DEGs and 805 downregulated DEGs, while there were 910 DEGs between the ZnO group and the control group, including 263 up-regulated DEGs and 647 down-regulated DEGs. In addition, a total of 3481 transcripts were differentially expressed after treatment with ZnO NPs, of which 1670 DETs were upregulated and 1811 DETs were downregulated, while 3323 transcripts were significantly different when under ZnO exposure, showing that 1489 DETs rose and 1834 DETs fell (Figure 4C,D). The results indicated that the quantity of DEGs and DETs of the ZnO NPs group was more than in the ZnO group, and the overall differences in genes or transcripts in the ZnO NPs group were more pronounced than in the ZnO group, which reflects the complex impacts of the nano-state on the toxicity of ZnO compounds. As shown in the Venn diagram (Figure 5A–C), a total of 1420 cellular processes affected by the ZnO NPs and ZnO were analyzed, of which 51.27% (728 cellular processes) were affected by both of the above exposures. In total, 510 and 182 processes were unique to ZnO NPs and ZnO treatments, accounting for 35.92% and 12.82% of the total, respectively. In addition to 233 common up-regulated processes, ZnO NPs and ZnO brought 200 and 30 unique upregulated changes, respectively. In addition, 495 downregulation processes were identified for ZnO NPs and ZnO, with 310 and 152 unique processes, respectively. Thus, the same cellular processes in the liver may be affected by both ZnO NP and ZnO exposure, which may be related to the fact that both substances were chemically derived from ZnO. However, their individual special influence processes were also represented; the sphere of influence triggered by ZnO NPs was wider than that of ZnO, due to the specific toxic effect of nano properties. In order to confirm the involvement of DETs among different samples in biological processes (BP), cell components (CC) and molecular functions (MF), GO annotation was analyzed, as shown in Figure 6. The evidence suggested that the differences in transcripts were mainly focused on BP and CC. According to the DET number compared to the control group, the two treatment groups have a strong consistency in enrichment items, such as biological regulation, the immune system process, responses to stimulus, membrane parts, and so on. However, they differ in the level of upregulated or downregulated DETs for certain functions, including biological mineralization and cell killing (Figure 6A,B). The DETs of ZnO NPs and ZnO were further compared directly, as shown in Figure 6C. In accordance with the principle that the number of upregulated and downregulated genes accounts for more than 10% of DETs, we found that the DETs in BP showed the main enrichment effect in biological regulation, the immune process, localization, metabolic processes, response to stimulus and signal-related process. Moreover, the DETs related to CC were enriched in the intracellular and extracellular regions, membrane parts, organelles and protein-containing complexes, while, in MF terms, the DETs were mainly associated with binding function and catalytic activity. It was noteworthy that the enrichment of ZnO NPs in the cell-killing process, cell detoxification and antioxidant function was significantly different from that of ZnO. Most importantly, the ZnO NPs group enhanced the cell killing and decreased the detoxification ability. The tests revealed that the toxicity mechanism of ZnO NPs and ZnO was partly different, and the Go terms implicated the serious effects of ZnO NP stimulation on membrane function, antioxidant function, cell killing and detoxification.

### 2.3. Cell Inactivation Caused by ZnO NPs and ZnO

**Figure 7:**
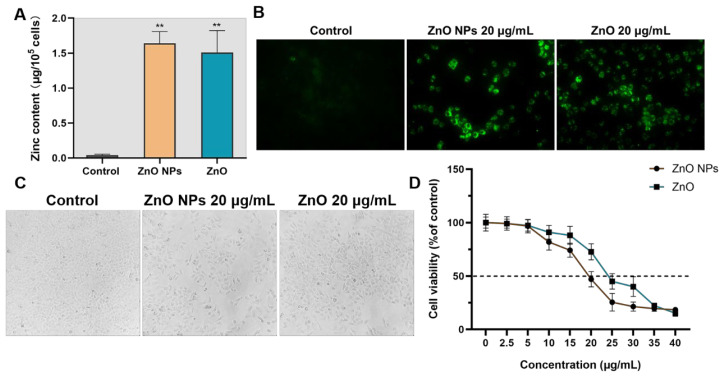
Cell inactivation, caused by ZnO NPs and ZnO. (**A**) Determination of Zn content in the HepG2 cells treated with ZnO NPs and ZnO by FAAS. (**B**) A TSQ fluorescent probe was used to detect the content of Zn2+ in HepG2 cells. (**C**) HepG2 cells treated with ZnO NPs and ZnO for 24 h were observed under an inverted light microscope (200×). (**D**) Cell viability of the HepG2 cells was measured after treatment with different concentrations of ZnO NPs and ZnO via a CCK-8 assay. Data represent the mean ± SD from three independent experiments. ** *p* < 0.01, compared to the control group.

In addition to the measurement of Zn ion content in an extracellular environment, the intracellular accumulation of Zn ions was also evaluated. As shown in Figure 7A,B, the 20 μg/mL ZnO NPs and ZnO represented similar levels of Zn^2+^ concentration to HepG2 cells after 24-h exposure. Considering the stability characterization in 3.1, this result suggests that the acidic environment, such as with cellular lysosomes, may provide an auxiliary for ion transformation from ZnO NPs to Zn ions, although ZnO exhibited a stronger ability to release Zn ions than ZnO NPs at different concentrations and times. Then, we found that the exposure of ZnO NPs and ZnO increased the intercellular space in Figure 7C, while cell viability was reduced through incubation with both treated groups in a dose-dependent manner (Figure 7D). However, the ZnO NP-induced cell loss was more remarkable than in ZnO, and the IC_50_ under exposure to ZnO NPs and ZnO was approximately 20 and 25 ng/ml, respectively, implying more lethal cell depletion being triggered by ZnO NPs, compared with ZnO when given the same amount of the Zn element.

### 2.4. Comparison of Cellular Oxidative Stress

**Figure 8:**
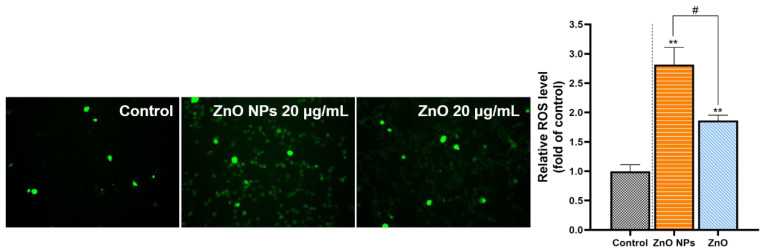
Comparison of cellular ROS. A DCFH-DA fluorescent probe was used to detect ROS accumulation in HepG2 cells after treatment with 20 μg/mL ZnO NPs and ZnO for 24 h. Images were observed under a fluorescence microscope (200×), and the fluorescent intensity was analyzed using the ImageJ software. Data represent the mean ± SD from three independent experiments. ** *p* < 0.01, compared to the control group; ^#^ *p* < 0.05, compared to the ZnO NP group.

Predicting the oxidative stress damage that could occur in HepG2 cells according to the transcriptome analysis, ROS accumulation was evaluated in our research. The results showed that the fluorescence intensity was obviously enhanced through exposure to ZnO NPs and ZnO. Based on the calculation, the ZnO NPs-induced increase of ROS accumulation was significantly higher than that in the ZnO group. The result suggested that nanometer-sized ZnO could enlarge the impact on the status of ROS (Figure 8).

### 2.5. Mitochondria Damage Triggered by ZnO NPs and ZnO

**Figure 9:**
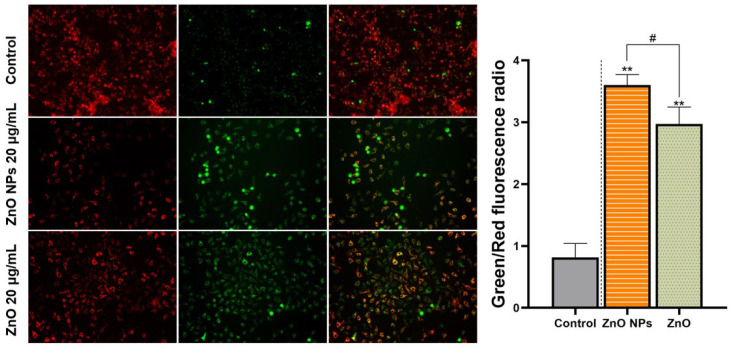
Mitochondria damage, triggered by ZnO NPs and ZnO. (A) HepG cells were treated with 20 μg/mL ZnO NPs and ZnO for 24 h; the MMP was detected by JC-1 staining. Red shows the aggregate form of JC-1, representing the normal MMP; green shows the monomer form of JC-1, representing the decreased MMP (200×). Data represent the mean ± SD from three independent experiments. ** *p* < 0.01, compared to the control group; ^#^ *p* value = 0.0281, compared to the ZnO NPs group.

After the stimulation of ZnO NPs and ZnO, the extent of mitochondrial damage was tracked by the lost red fluorescence and increased green fluorescence in HepG2 cells. This demonstrated that the MMP was decreased by these two different Zn sources, in terms of the increased ratio of green fluorescence to red fluorescence in the field of view of the microscope. Furthermore, as shown in Figure 9, the change of MMP under ZnO was not as serious as the influence of ZnO NPs. The above results verified that mitochondrial permeability was sensitive to ZnO NPs.

### 2.6. The Different Levels of Ca^2+^ Accumulation

**Figure 10:**
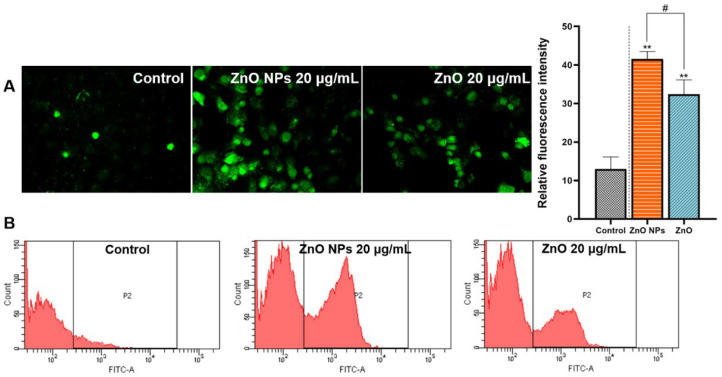
The level of Ca^2+^ accumulation. HepG2 cells were treated with 20 μg/mL ZnO NPs and ZnO for 24 h. A Fluo-3 AM fluorescent probe was used to detect Ca2+ accumulation. (**A**) Images were observed using a fluorescence microscope (200×); the fluorescent intensity was analyzed using ImageJ software. (**B**) Ca^2+^ accumulation was detected by flow cytometry. Data represent the mean ± SD from three independent experiments. ** *p* < 0.01, compared to the control group; ^#^ *p* < 0.05, compared to the ZnO NP group.

ZnO NPs/ZnO-induced cellular Ca^2+^ accumulation was measured by the intensity of green fluorescence. After further quantification, the total Ca^2+^ in the HepG2 cells, incubated with ZnO NPs and ZnO, was certified to be dramatically increased, compared with the control group. It also revealed the effect of ZnO NPs on the green fluorescence, which shows that the Ca^2+^ levels were higher than in the ZnO group (Figure 10A). Flow cytometry further found more Ca overload-positive cells in the group exposed to ZnO NPs (Figure 10B).

### 2.7. Effects on Biochemical Indices and Antioxidant Levels in a Rat Model

**Figure 11:**
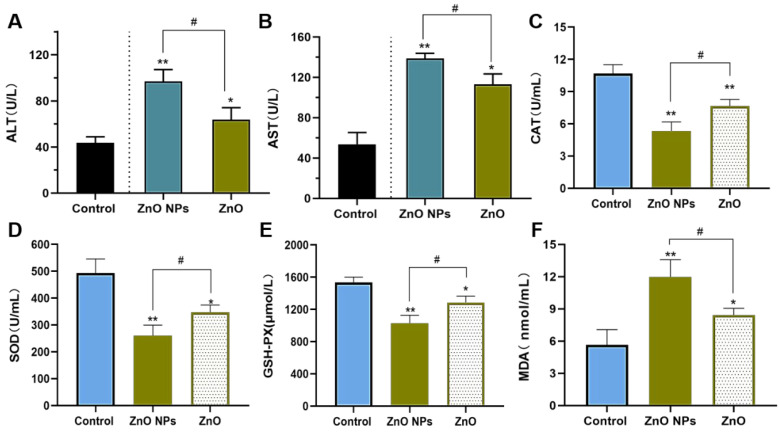
Biochemical indices and antioxidant levels in a rat model. The rats were intraperitoneally injected with the ZnO NPs group (25 mg/kg) and ZnO group (25 mg/kg) for one week. (**A**) ALT serum levels. (**B**) AST serum levels. (**C**–**F**) The effect of ZnO NPs and ZnO on CAT activity, SOD activity, GSH-PX activity and MDA levels in the serum of the rats. Data represent the mean ± SD (*n* = 6). * *p* < 0.05, ** *p* < 0.01, compared to the control group; ^#^ *p* < 0.05, compared to the ZnO NPs group.

As exhibited in Figure 11A,B, the enzymes in the plasma displayed a consistent trend of changes in the two treated groups. The plasma of the rat model stimulated by ZnO NPs and ZnO showed a higher index of hepatic function while the content of AST and ALT that was increased by ZnO NPs was significantly higher than that of the ZnO group. Moreover, compared with ZnO, ZnO NPs-mediated lipid peroxidation in the liver was more obvious, and the reduction of antioxidant capacity was more serious, suggesting that ZnO NPs greatly contributed to the generation of liver toxicity (Figure 11C–F).

### 2.8. Comparison of Rat Liver Damage Induced by ZnO NPs and ZnO

**Figure 12:**
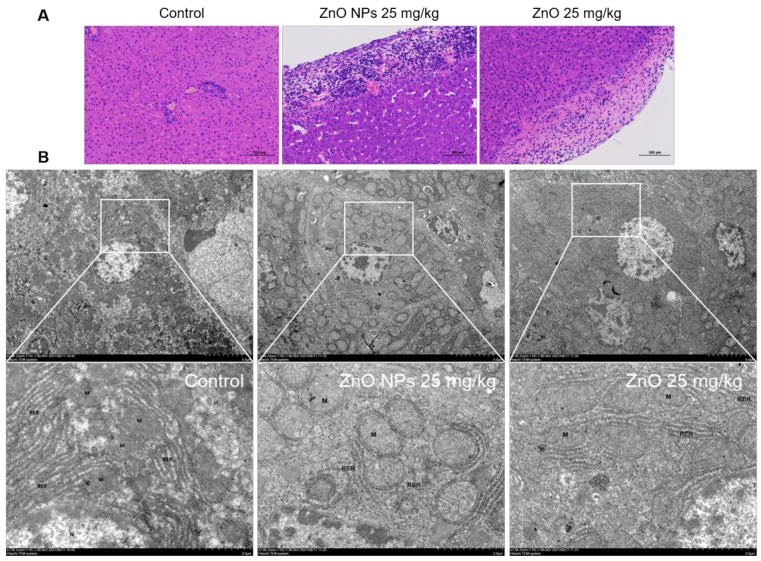
Comparison of rat liver damage induced by ZnO NPs and ZnO. (**A**) Histopathology of HE-stained liver sections from ZnO NP and ZnO-treated rat tissue (Bar = 100 μm). (**B**) Effect of ZnO NPs and ZnO on ultrastructure changes in rat liver, as observed with an electron microscope (Bar = 5 or 2 μm).

Compared to the typical liver structure shown by the healthy group, the rat samples in all the other treatment groups showed tissue damage to the liver. As shown in Figure 12A, cell disorder and intercellular hyperemia occurred in both the ZnO NPs group and ZnO group. Microscopically, groups of ZnO NPs and ZnO produced similar inflammatory damage to the mouse liver, while the inflammatory cells’ infiltration and decreased intercellular compactness, which were induced by ZnO NPs, were more obvious and the ZnO NPs induced lesions that spread more widely into the tissue. Furthermore, the microstructural damage of hepatocytes was also determined via TEM (Figure 12B). In the rat liver exposed to ZnO NPs, the nucleus was pyknotic, the endoplasmic reticulum was incomplete, the mitochondrial crest was broken and the shape of the mitochondria was transformed into swollen spheres, whereas the mitochondrial enlargement and the karyopyknosis under ZnO exposure was not as serious as in the ZnO NPs group.

## 3. Discussion

The reason for the selection of ZnO NPs and ZnO as the emphasis of our study was that with the continuous improvement of application materials in the fields of everyday chemicals, agriculture, medicine or therapies, ZnO NPs that are transformed from ZnO particles demonstrate increasing contact with to human beings; their contact with live bodies is more extensive compared to that in the past [2,44,45]. It is important to underline the necessity of toxicological research on ZnO NPs, which arise from the usefulness they offer in applications related to humans and animals [38,46,47]. Compared with ordinary ZnO, the particle size of ZnO NPs is more useful and the value of the specific surface area and the surface atomic number is higher, with unsaturated characteristics and strong biological activity [48]. Meanwhile, because of the small diameter, the passive diffusion of ZnO NPs was easily facilitated, further reducing the consumption of energy, the ligand and the carrier, increasing interaction sites with tissue. In the case of the previous literature on animal applications, despite the fact that the biological activities of ZnO NPs may increase the toxicity risk of the original ZnO, their application value and research status were also affected. The antibody titer of sheep red blood cells was increased more significantly in laying hens that were fed diets supplemented with 80 mg/kg ZnO NPs, compared to the ZnO-treated hens, indicating that ZnO NPs showed better improvement in terms of the immune system’s defense function [49]. In addition, although both 500 mg/kg ZnO NPs and a high dose of conventional ZnO reduced the ileum crypt depth and promoted the growth of piglets, the former exhibited better anti-inflammatory effects and higher upregulation levels of TFF3 and Nrf2 in the duodenum and jejunum than the latter [50]. In another experiment, 450 mg/kg ZnO NPs and 3000 mg/kg ordinary ZnO showed similar effects on daily weight, intestinal villus and the diarrhea rate in piglets, suggesting that low doses of ZnO NPs could achieve the same effect as high doses (pharmacological doses) of ZnO [51]. Similarly, the advantages of 800 mg/kg ZnO NPs and 3000 mg/kg ZnO were compared; the intestinal villi in the ZnO NPs group were more improved and the plasma diamine oxidase was more significantly inhibited than that in the ZnO group. Moreover, the expression levels of the tight junction proteins, IL-1β and TNF-α, increased more significantly [52]. It is most noteworthy that, compared with a combination of 20 mg/kg colistin sulfate and 3000 mg/kg ZnO, the 1200 mg/kg ZnO NPs displayed an equivalent improvement in body weight and the incidence of diarrhea, with comparable effects on serum enzymes and tight junction proteins, in an evaluation using a weaned piglet model [53]. Together, the application potential of low-dose ZnO NPs, a substitute for ZnO, emerged in various fields, including medicine, food and feed. Although our assessment was confined to the detection of the particles’ toxicological properties, the data could further assist us with the explanation of the higher applied rate of ZnO and determine the research significance.

It was well-known that the toxic effects of ZnO NPs were related to their nano properties [41]. In addition, the ZnO NPs appeared different physicochemical properties including size, shape, specific surface area, surface modification, protein crown binding and electrostatic interaction compared with traditional ZnO [31]. In order to analyze the toxicity of ZnO NPs based on physicochemical features, we roughly characterized the ZnO NPs (Figure 2A–D). In this test, we found the unique physical and chemical properties of the ZnO NPs. In routine culture solution, we also confirmed that the level of Zn ion release from ZnO NPs was lower than that from traditional ZnO (Figure 3A,B). However, ZnO NPs released Zn ions more fully in acidic conditions (pH ≤ 5.5) (Figure 3C), leading to similar intracellular Zn content to the ZnO group (Figure 7A,B). There were some points where the Zn ion release was one of the factors affecting the toxicity induced by the Zn compound, while our investigation demonstrated that Zn ion release and intracellular Zn ion accumulation generated by ZnO NPs was not stronger than that by ZnO itself. One caveat with another trial is that the existing evidence suggests that although dissolved Zn ions were the first to trigger toxicity in mice alveolar, the disparity of toxicity induced by different ZnO NPs mainly depended on the action of insoluble particles [54]. Here, the most intuitive difference between ZnO NPs and conventional ZnO in the literal sense was the difference between nano and non-nano sizes, that is, the difference between nano-sized ZnO and micro-sized ZnO and the difference in size (specific surface area) between them. Numerous pieces of evidence also viewed size as key to adjusting the toxicity of ZnO NPs. The dopaminergic neuron toxicity raised by ZnO NPs, with diameters of 50 nm and 100 nm, was more serious than that by 1000 nm ZnO NPs, due to the different ability of BBB penetration and cellular absorption [55]. The reduction of the survival rate caused by ZnO NPs at 26 nm was more severe than 78 and 147 nm in human lymphoblastic cells. Similarly, mice alveolar macrophages were exposed to 10–30 nm and 100 nm ZnO NPs, resulting in IC_50_ levels of 26.74 and 47.37 g/mL, respectively [54]. It was speculated, however, that pony-sized ZnO NPs were more toxic than large particles, merely at low concentrations, whereas they may present an equal level of risk at high concentrations, possibly due to the tendency of pony-sized nanomaterials to agglomerate [56]. In addition to cell viability indicators, the teratogenic potential was also mainly reflected in the ZnO NPs with a small diameter, which could lead to the emergence of a micronucleus in the LT97 cell at 50 nm, not at 100 nm [57]. As is consistent with these comparative tests, here, the RNA sequencing clarified that compared with ordinary ZnO, ZnO NPs demonstrated stronger lethality to hepatocytes, brought more severe inhibition of cell detoxification and caused more interference with antioxidation processes and membrane function (Figure 4, Figure 5 and Figure 6). Consistent with RNA sequencing, in vitro experiments determined that ZnO NPs evoked more cell loss (Figure 7C,D) and ROS accumulation (Figure 8) than ZnO, as well as mitochondrial membrane potential decline (Figure 9). Besides, as one of the mechanisms of mitochondrial dysfunction, intensified calcium overload was also disclosed in the ZnO NPs group (Figure 10). Coincidentally, another document verified that intracellular Zn ions mainly influenced the activity of enzymes and transcription factors, while the effects of ZnO NPs focused on the Ca^2+^ flux, ROS generation, membrane damage and mitochondrial dysfunction [39]. In our rat model, ZnO NPs disrupted liver function more significantly than ZnO particles (Figure 11A,B). Furthermore, the pathological changes and oxidative stress damage (Figure 11C–F) were more significant (Figure 12A,B). To summarize, on the basis of the principle that the dosage of Zn-derived additive in practical applications was generally stipulated and calculated by Zn element concentration, we proved that ZnO NPs exhibited more serious hepatocellular toxic effects than traditional ZnO under the consistent concentration of the Zn element, as presented in the predicted molecular mechanisms in Figure 1.

Although we preliminarily elucidated the hepatotoxic mechanism of ZnO NPs and ZnO and gave the results of a comparative evaluation, the limitations of this study should not be ignored. Past data showed that the risk of ZnO NPs fluctuated in different biological models, and the toxicity was not always uniformly affected by the different properties of ZnO NPs. As one case demonstrated, the toxic effects of large ZnO nanorods on human lung epithelial cells were more significant than small ZnO NPs [58]. Here, HepG2 cells and rat models were used to determine the difference between the hepatotoxic effects of ZnO NPs and ZnO, whereas the differences identified in this study may not be applicable to other toxicological models. Furthermore, ZnO NPs could also be compared with other Zn-derived compounds since ZnO was not the exclusive donor of Zn element. Through the methods of subcellular fractionation from crucian carp liver, the differences in detoxification and distribution between 2 mg/L ZnO NPs and Zn ions were found in another study [59]. It was also reported that damage to zebrafish embryos, the vascular system and dorsal root ganglion induced by the stimulation of ZnO NPs (1-100 μg/mL) was more obvious than that of other dissolved Zn ions [60]. Therefore, all other soluble Zn sources were suitable as an object of comparison with ZnO NPs. The latest investigations suggested that ZnO NPs caused more mitochondrial dysfunction than ZnCl_2_ [41]. The contents of Na^+^ and K^+^ in the spleen and thymus of mice exposed to ZnO NPs were higher than in the ZnP group, and the effects of ZnO NPs on ion homeostasis and immunity were more significant [61]. After treatment with ZnO NPs and ZnSO_4_ via nasal drips, the Zn element was found to accumulate in the liver only in the ZnO NPs group [62]. In conclusion, the above findings encourage that future comparative studies of ZnO NPs should be extended to other toxicological models and other Zn-derived compounds, to gain a more complete understanding of the changes in toxicity of nano-upgraded compounds.

In conclusion, our current research emphasized the increased toxicity of ZnO NPs despite the recognition of their application value. Both the ZnO NPs and ordinary non-nanoscale ZnO were able to produce liver toxicity in vivo and in vitro with equal amounts of the Zn element. However, in the transcriptional level, ZnO NPs disturbed the hepatocyte more perniciously. Compared with ZnO, the rat liver damage induced by ZnO NPs was more significant, and the oxidative stress, mitochondrial damage, and intracellular calcium overload induced by ZnO NPs were more serious than in ZnO. Therefore, ZnO NPs may enhance the hepatotoxicity of conventional ZnO, which facilitates our understanding of the liver toxicity mechanisms of Zn-derived compounds in different states. The results were also advantageous for providing a more comprehensive risk assessment of ZnO NPs in terms of future human-accessible applications, especially clinical treatment, biomedicine, agricultural additives and household products. The more we understand the drawbacks of the nano-modification of ZnO, the safer it will be to use it to upgrade technology and life.

## 4. Materials and Methods

### 4.1. Reagents and Chemicals

The ZnO NPs (20 nm–50 nm, >98% pure) and ZnO used in the studies were acquired from Sigma-Aldrich (St. Louis, MO, USA), and were stored at 4 °C. Dimethyl-sulfoxide (DMSO), Fluo-3AM (fluorescent probe of Ca^2+^) and JC-1 (probe of mitochondrial membrane potential) were purchased from AMRESCO Inc. (Solon, OH, USA). A cell counting Kit-8 (CCK-8) and 2′,7′-dichlorofluorescensin diacetate (DCFH-DA) were obtained from Solarbio Science & Technology Co., Ltd. (Beijing, China). Cell culture reagents, including Dulbecco’s Modified Eagle’s medium (DMEM) and fetal bovine serum, were provided by Life Technologies (Therma Fisher Scientific, NY, USA) and Tianhang Co., Ltd. (Deqing, China). Western blotting reagents were acquired from the Beyotime Institute of Biotechnology (Haimen, China). An assay kit of oxidative stress species was obtained from Jiancheng Bioengineering Inc. (Jiangsu, China). The gluta solution was provided by the Aladdin Company (Shanghai, China). Sodium carboxymethylcellulose and Formalin Fix Solution (10%) were purchased from Solarbio Science & Technology Co., Ltd. (Beijing, China).

### 4.2. ZnO NPs Characterization and Quantitative Analysis

The white ZnO NP powders were filtered and dissolved in deionized water (Barnstead E-pure), followed by sonication in ultrasonic cleaners (UCM-6S, KUANSONS, Shanghai, China) for 20 min. Field emission TEM (Technai G2 S-TWIN, Fei, Hillsboro, OR, USA) was applied to analyze the morphological properties of ZnO NPs. An intelligent Raman spectrometer (DXR3, Thermofisher Scientific, Waltham, MA, USA) was used to observe the chemical compositions. The dispersibility, polymerizability, hydrodynamic diameter and zeta potential were presented through the conduction of dynamic light scattering (DynaPro NanoStar, Wyatt, Santa Barbara, CA, USA) with a zeta potential analyzer (Nano-ZS, Malvern, UK). The contents of Zn^2+^ released from ZnO NPs and ZnO were quantitatively evaluated by flame atomic absorption spectroscopy (FAAS, ZA3000, HITACHI, Beijing, China).

### 4.3. Animal Exposure

The Vital River Animal Technology Co., Ltd. (Beijing, China) provided eighteen healthy male Sprague-Dawley rats at 8 weeks old (180–200 g), which were subsequently divided into a control group, a ZnO NP group (25 mg/kg) and ZnO group (25 mg/kg) after we received the shipment, followed by a one-week acclimation. All the rats were given fresh water and commercialized feed. We maintained six rats per plastic cage, with the animal facility kept at 35 ± 10% of humidity, at 22 ± 3 °C, and with suitable air circulation in a light-dark cycle (12 h/12 h). All groups suffered continuous intraperitoneal injection with access to standard breeding conditions for seven days. All the operations involved in the animal experiments were reviewed and approved by the Animal Welfare and Ethics Committee of Nankai University, and the approval number of the commission is 2022-SYDWLL-000415. All testing procedures and arrangements were designed to minimize animal suffering.

### 4.4. Histopathological Detection

Seven days after the administration, the liver was dissected from the rats. Then, the isolated tissue was fixed in formalin buffer (10%) overnight. After that, the sample was prepared in small pieces as appropriate, then treated with alcohol, xylene and paraffin. The hepatic paraffin blocks obtained in the last step were converted into thin paraffin sections (5–8 mm) using a rotary microtome (RWD, Shenzhen, China). Following this step, the preprepared paraffin slices were stained with hematoxylin and eosin (H&E) and subjected to histopathological detection under a light microscope (Primotech, Beijing, China) to observe the level of abnormal architecture in the control, ZnO NPs and ZnO groups.

### 4.5. Ultrastructural Analyses

Ultrastructural observation, involving plasma membrane rupture and mitochondrial damage, was completed using a JEM-1011 electron microscope (Tokyo, Japan). The tissue and cell samples were harvested after rinsing 3 times with PBS. All samples were soaked in ice-cold glutaraldehyde (2.5%, KERST, Qingdao, China) for 3 hours. In addition, the samples were incubated with osmium tetroxide; after 1 h, ethanol series were applied for sample dehydration. Thereafter, the specimens were placed in epoxy resin for 12 h. Uranyl acetate and lead citrate were used to treat the ultrathin slices the next day, followed by morphological observation via the electron microscope mentioned above.

### 4.6. Liver Function Indicator and Oxidative Stress Index

The serum was collected in the supernatant after a 4 °C centrifugation of the whole blood, followed by instant testing of AST and ALT, achieved with an automatic biochemical analyzer (PUZS 300A/X, Perlong, Beijing, China). Moreover, the expression levels of MDA, superoxide dismutase (SOD), glutathione peroxidase (GSH-PX) and CAT were evaluated using the corresponding kit products (Lipid Peroxidation MDA Assay Kit, WST-8 Total SOD Activity Detection Kit, Total GSH Activity Detection Kit and CAT detection kit, Biotechnology, Haimen, China). The detection absorbance of the above oxidative stress indicator was set as 535 nm, 450 nm, 410 nm and 240 nm, under a microplate reader (absorption light microplate reader, 800TS, Biotek, Winooski, VT, USA). After reading, the levels of antioxidant and oxidative stress products were estimated, according to the relevant standard curve.

### 4.7. Cell Culture

Human hepatocellular carcinoma cell lines (HepG2 cells, obtained from ATCC) were utilized as a trial model of ZnO NPs and ZnO infection in vitro. We used DMEM supplemented with 10% serum and 1% penicillin-streptomycin mixture (HyClone SV30010 10,000 U/mL, Cytiva, Shanghai, China) as the cell culture medium. It was necessary to keep the temperature at 37 °C and the CO_2_ level at 5%. Every third day, the Petri dishes were updated for cell passage. During the logarithmic growth phase of the HepG2 cells, 20 μg/mL ZnO NPs were used as a primary interferer for 24 h. For comparison of the toxic effects, ZnO was also used to stimulate cells with equal amounts of the Zn element.

### 4.8. Cell Viability Examination

The viability of HepG2 cells was assessed using the CCK8 method. Briefly, the cells were seeded in a 96-well plate and cultured for 24 h to a density of 2 × 104 cells/well. Then, the cells in the control group were exposed to 0.2% DMSO, while other groups were treated with ZnO NPs and ZnO, with a gradient-increased concentration. After one night, we discarded the culture medium and incubated the cells using 10% CCK8 (Solarbio Science & Technology, Beijing, China), according to the operation protocols, for 30 min, keeping them out of the light in a 37 °C incubator. After this period, the impact of ZnO NPs and ZnO on the cell survival level was measured via a microplate reader (absorption light microplate reader, 800TS, Biotek, Winooski, VT, USA) at a wavelength of 450 nm. The higher the absorbance value, the higher the viability and the less the influence of the stimulus.

### 4.9. Oxidative Stress Measurement

For the evaluation of ROS production, HepG2 cells were incubated in 12-well plates and maintained in this state for 24 h. Following this step, every well was exposed to 20 μg/mL ZnO NPs and ZnO overnight. Thereafter, 2, 7-dichlorofluorescein diacetate (DCFH-DA fluorescence probe, Solarbio Science & Technology, Beijing, China) was utilized as a substitute for the cell culture medium. After 30 minutes of oxidation reaction, the fluorescence intensity, which represents the amount of DCF in cells that underwent oxidative stress, was observed and counted under an inverted fluorescence microscope (LF500, Laite, Guangzhou, China) at 530 nm. The goal for comparison was the percentage alteration of positive cells.

### 4.10. Mitochondrial Staining

A JC-1 Assay Kit (AMRESCO, Solon, OH, USA) was used to investigate the variation of MMP induced by ZnO NPs and ZnO. After a cell exposure test in 12-well culture plates, the JC-1 indicator solution served as a substitute for the cell culture medium, followed by 30 minutes of staining, according to the manufacturer’s description. According to the detection principle, normal mitochondrial membrane potential could exhibit aggregates of JC-1 in mitochondria and emit red fluorescence. Conversely, the potential drop could lead to the release of JC-1, which further increased the green fluorescence on behalf of the JC-1 monomer. The loss of mitochondrial membrane potential was visualized through the ratio of green to red fluorescence under the aforementioned Laite fluorescence microscope.

### 4.11. Ca^2+^ Flow Assessment

For monitoring the intracellular Ca^2+^, the HepG2 cells grown in the 96-well plates overnight were followed by exposure to the ZnO NPs and ZnO. The changes in intracellular Ca^2+^ levels were determined through a calcium kit (Fluo-3AM) and followed the steps of the instruction manual. Afterward, the Ca^2+^ response was reflected in the value of relative fluorescence intensity at 488 nm–506 nm. The intracellular Ca^2+^ was also quantified by flow cytometry. Briefly, after 24 h of exposure to ZnO NPs and ZnO with the same concentration, the HepG2 cells were digested and resuspended in PBS. Afterward, Fluo-3-AM staining was applied under the guidance of the manufacturer’s instructions, and the flow cytometry detection system (FACSCanto II, Becton Dickinson, COA, USA) was applied to measure the number of cells with entered fluorescence as a required procedure and was represented via a quadrant diagram.

### 4.12. RNA-Sequencing (RNA-seq) Analysis

After being pretreated by different zinc sources for 24 h, the HepG2 cells of both the control group and testing groups were digested by pancreatin and harvested using centrifugation (1500 rpm, 2min). Subsequently, the cells were mixed with Trizol buffer on ice for 5 min, followed by storage in a nitrogen canister. The total RNA was prepared using an RNA Extraction Kit (Invitrogen, Carlsbad, CA, USA) as soon as the cells were resuscitated, followed by quality control with a bioanalyzer (Agilent, Santa Clara, CA, USA). Subsequently, the ScriptSeq™ Complete kit (Epicentre, Madison, WI, USA) was applied to the construction of the RNA-sequencing library. The messenger RNAs (mRNAs) sequencing was carried out following the protocol with a Hiseq 4000 instrument (Illumina, San Diego, CA, USA) from the Origin-gene company (Origin-gene, Shanghai, China), with three replicates of each sample. The significant differences between the control and testing groups on mRNA levels were provided for further analysis. The transcriptome-sequencing data has been deposited in the Sequence Read Archive (SRA) database of NCBI (https://www.ncbi.nlm.nih.gov/sra, accessed on 6 June 2022) under the SRA accession ID SRP375919 (data release date: 6 June 2022).

### 4.13. Statistical Analysis

All independent experiments were repeated no less than three times. Each data point was carried out as a mean, accompanied by standard deviations (SDs), in GraphPad (Prism 8, USA). Statistical processing and comparative analyses were completed according to the principle of a one-way analysis of variance (ANOVA). The gap in the data among ZnO NPs and ZnO was judged to be significant if *p* ≤ 0.05.

## Figures and Tables

**Table 1:** Overview of recent toxicity studies of ZnO NPs with different models.

ZnO NPs Dosage	Toxicity Classification	Species/Cell Type	Exposure/Assay	Observations	Ref.
0.05, 0.2 mg/kg	Acute toxicity	Mice	Single intravenous exposure	Accumulation to spleen, liver and lungs; increase of 8-OHdG. LD50: 0.3 mg/kg	[5]
9.38, 18.75, 37.50, 75.00, 150.00 mg/kg	Acute toxicity	Mice	Intraperitoneal injection for 5 d and 10 d	Histopathological damage to kidneys, spleen, heart, and brain; effects on hematological and biochemical parameters. LD50: 299.9 mg/kg	[6]
31.25, 125, 500 mg/kg	Subchronic toxicity	Rat	Oral gavage for 90 d	Inflammatory damage to stomach, pancreas, eye, prostate gland tissues.	[7]
2000 mg/kg	Teratogenicity	Rat	Oral gavage	Fetal malformation, skeletal dysplasia and spinal insufficiency.	[8]
0.245, 245.26 mg/kg	Mutagenicity	Chicks	Intraperitoneal injection for 2 d	Induction of micronucleus, binucleus and heteromorphus in erythrocytes.	[9]
1 µg/mL	Carcinogenic risk	Mice embryonic fibroblasts	Exposure for 2 and 12 weeks	Upregulation of the early biomarkers of carcinogenesis, MTH1.	[10]
10 mg/kg	Nephrotoxicity	Mice	Single intraperitoneal injection	Renal tubule and glomerulus damage, increase of serum creatinine and blood urea nitrogen; HIF-1α-mediated apoptosis and autophagy.	[11]
600, 1000 mg/kg	Cardiotoxicity	Rat	Oral gavage for 5 d	Increase of troponin-T, creatine kinase-MB, myoglobin, TNF-α, IL-6, cardiac calcium concentration, DNA damage and Caspase-3 activity.	[12]
5.6 mg/kg	Neurotoxicity	Mice	Intraperitoneal injection 3 times per week for 28 d	Damage to blood brain barrier; disorder of nerve cell arrangement; neuronal degeneration; nistenite loss.	[13]
350 mg/kg	Immunotoxicity	Rat	Oral gavage for 28 d	Increase of MDA, IL-1β, TNF-α, TLR4 and TLR6; increase of apoptotic bodies and tingible body macrophages; appearance of abnormal thymocytes;	[14]
100 mg/kg	Reproductive toxicity	Mice	Oral gavage for 3 d	Induction of oxidative stress and apoptosis; weight loss of male mice testicular; activation of Shh pathway in ovaries; Caspase-related uteri injury.	[15]

## Data Availability

Data in the current study are available from the corresponding author on request.
